# Spermine oxidase promotes oxidative stress during islet inflammation

**DOI:** 10.1016/j.jbc.2026.113412

**Published:** 2026-08-07

**Authors:** Christian Checkcinco, Batoul Hammoud, Xia S. Saavedra, Armando A. Puente, Hardik Shah, Sarah A. Tersey, Ryan M. Anderson, Raghavendra G. Mirmira

**Affiliations:** 1 Department of Medicine and the Diabetes Research and Training Center, The University of Chicago, Chicago, Illinois, USA; 2 Metabolimics Platform, Comprehensive Cancer Center, The University of Chicago, Chicago, Illinois, USA

**Keywords:** islet, polyamine, reactive oxygen species (ROS), inflammation, zebrafish

## Abstract

Diabetes is characterized by impaired glucose homeostasis resulting from dysfunction and loss of insulin-producing islet β cells, with oxidative stress emerging as a contributor to β-cell injury. The polyamines (putrescine, spermidine, spermine) regulate cellular stress responses, and their metabolism can generate reactive oxygen species (ROS) and reactive aldehydes *via* catabolic enzymes such as spermine oxidase (Smox) and polyamine oxidase (Paox). Transcriptomic analyses of mouse and human islets exposed to proinflammatory cytokines revealed induction of the gene encoding Smox but no change in the gene encoding Paox, suggesting a role for Smox in amplifying inflammation-associated stress in islets. In developing zebrafish, the *smox* gene is expressed in the pancreas and enriched within endocrine cells. Using a β-cell-specific ROS-inducible zebrafish model, *smox* gene knockdown reduced intra-islet ROS and macrophage recruitment. Metabolomics of zebrafish embryos showed that Smox deficiency did not affect spermine or spermidine levels but led to accumulation of acetylated polyamines, consistent with the rerouting of polyamine flux through alternative acetylation pathways. RNA sequencing of zebrafish embryos demonstrated that Smox deficiency induces distinct transcriptional programs, with enrichment of cell cycle and RNA processing pathways at baseline and reprogramming of stress, translational, and immune-associated pathways during injury. Mechanistically, Smox is the primary polyamine pathway generating ROS in zebrafish, as reducing flux through the Paox-dependent pathway did not reduce ROS production. These findings support a model in which Smox amplifies β-cell stress through peroxide- and aldehyde-mediated toxicity and identify polyamine catabolism as a modifiable pathway in the setting of β-cell injury.

Pancreatic β cells play a central role in maintaining glucose homeostasis through regulated insulin secretion, and their dysfunction is a defining feature of both type 1 and type 2 diabetes. In type 1 diabetes, β-cell loss is driven by immune-mediated destruction, whereas in type 2 diabetes, β cells undergo progressive functional decline under metabolic stress. Despite differences in etiology, both conditions share common pathways of β-cell stress and dysfunction, including oxidative stress, endoplasmic reticulum stress, and impaired protein homeostasis (1). Among these, oxidative stress is particularly important, as β cells have limited antioxidant capacity and are therefore highly susceptible to reactive oxygen species (ROS)-induced damage (2, 3, 4). Elevated ROS levels can disrupt mitochondrial function, impair insulin biosynthesis, and promote the accumulation of misfolded or modified proteins, ultimately contributing to loss of β-cell function (5).

Polyamine metabolism has emerged as an important regulator of cellular stress responses and may contribute to oxidative stress within β cells. The polyamines, putrescine, spermidine, and spermine, are biogenic polycationic amines that regulate nucleic acid interactions, mRNA translation, redox balance, and cellular stress responses (6, 7). Their biosynthesis is tightly controlled through ornithine decarboxylase–dependent production of putrescine followed by sequential conversion to spermidine and spermine, while catabolic enzymes, including spermine/spermidine N^1^-acetyltransferase (Sat1), N^1^-acetylpolyamine oxidase (Paox), and spermine oxidase (Smox), regulate polyamine flux and turnover. Notably, polyamine catabolism can induce ROS, as these pathways generate hydrogen peroxide and reactive aldehydes. (8, 9). Smox and Paox directly couple polyamine catabolism to the production of hydrogen peroxide and reactive aldehydes. In particular, Smox directly catalyzes the conversion of spermine to spermidine, generating hydrogen peroxide and 3-aminopropanal, the latter serving as a precursor for the highly reactive aldehyde acrolein. Smox activity amplifies oxidative damage and inflammation in multiple tissues. In the gastric epithelium, *Helicobacter pylori*–induced Smox production promotes DNA damage and carcinogenesis risk (10), and increased Smox has been reported in prostate cancer and other inflammatory conditions (11). Additionally, in the mouse brain, *Smox* gene expression correlates with age, oxidative stress, and vulnerability of neurons to injury (12).

Although prior studies have shown that polyamine biosynthesis influences β-cell function under inflammatory stress, the specific contributions of Smox and polyamine catabolism to β-cell injury remain uninvestigated. Zebrafish provide several advantages for addressing these questions: their pancreatic composition, islet architecture, and key stress pathways are highly conserved with mammals; they are genetically and pharmacologically amenable; β-cell-specific ROS; and injury responses can be visualized and measured in a whole-animal context (13, 14). These features allow us to define developmental expression patterns, alter gene function, and measure metabolic and inflammatory responses *in vivo*. Based on Smox induction in inflammatory settings and its ability to generate reactive metabolites, we hypothesized that Smox contributes to β-cell pathology during inflammatory stress. In this study, we investigated Smox conservation, expression, and function in zebrafish pancreatic islets to define its contribution to β-cell stress and inflammatory injury.

## Results

### The gene encoding smox is conserved and expressed during zebrafish pancreatic development

**Figure 1:**
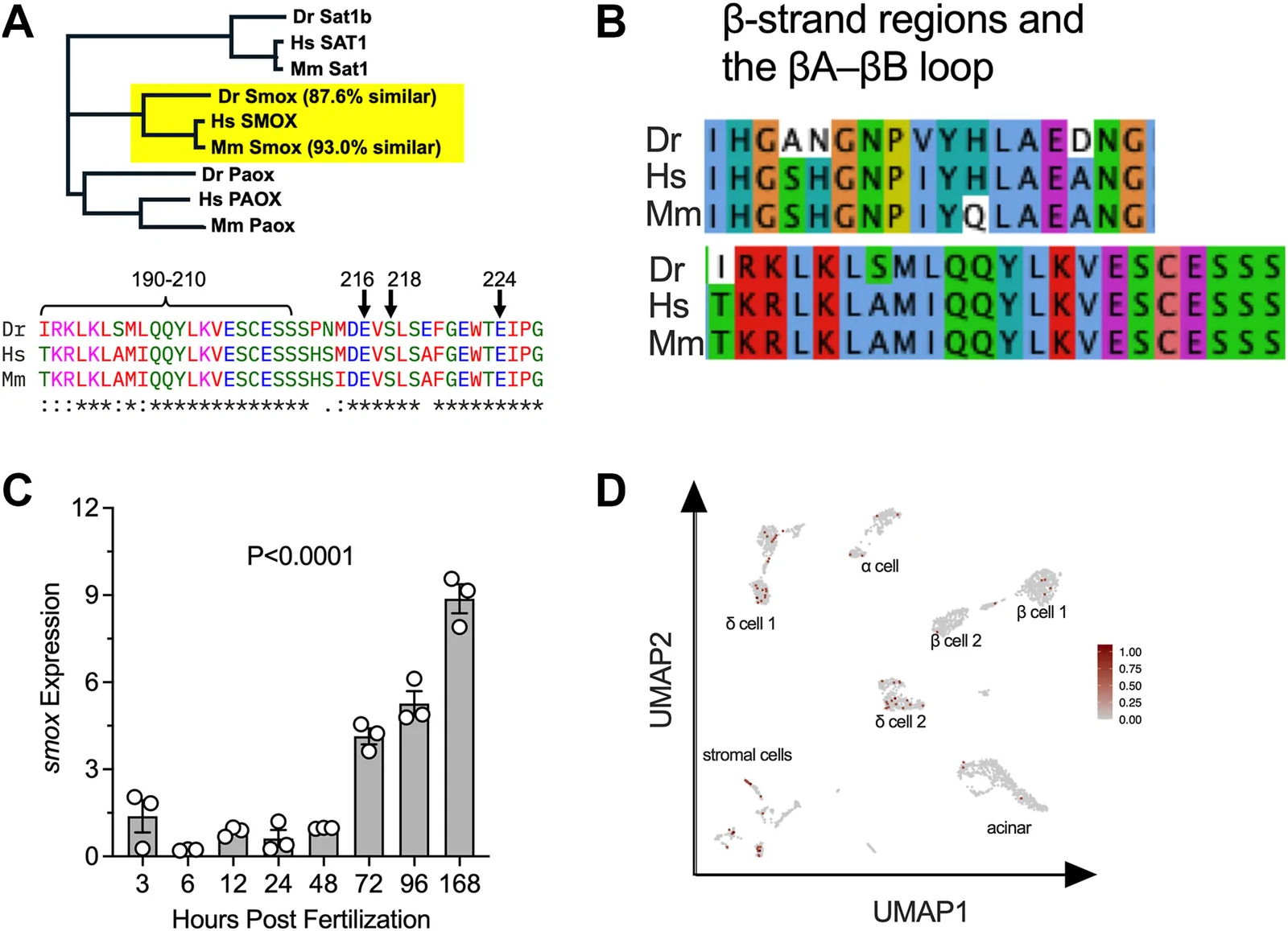
**Smox is conserved and expressed during zebrafish pancreatic development.***A*, phylogenetic tree demonstrating the evolutionary relationship of Smox across representative species. *B*, sequence alignment showing preservation of key structural regions, including the β-strands and the βA–βB loop, across Smox orthologs. *C*, developmental expression of *smox* in whole zebrafish embryos measured by quantitative PCR and normalized to *actb*. N = 3 biological replicates per timepoint. One-way ANOVA, no post-test. *D*, UMAP of *smox* expression across endocrine and exocrine populations in islets from adult zebrafish single-cell RNA-sequencing data. Data are presented as mean ± SEM.

To study Smox in the context of β-cell stress, we first assessed its protein sequence conservation across diverse species and the expression of the *smox* gene in the zebrafish pancreas. Phylogenetic analysis showed that the zebrafish Smox protein sequence clusters closely with other vertebrate orthologs, and sequence alignment demonstrated preservation of key structural motifs, including the β-strand regions and the βA–βB loop (Fig. 1, *A* and *B*). These features are consistent with prior work showing that polyamine-metabolizing enzymes maintain conserved domains important for catalytic activity (7, 8). Quantitative transcript analysis of zebrafish embryos revealed detectable expression of *smox* early after fertilization, followed by a notable increase at 72 h post-fertilization (hpf) (Fig. 1*C*). This rise at 72 hpf coincides with the establishment of pancreatic tissues and expansion of the principal islet, and this is a window in which polyamine pathways have previously been implicated in pancreatic organogenesis (15). Next, we analyzed a single-cell RNA-sequencing atlas derived from 2-month-old zebrafish islets (16) for *smox* expression. Transcripts for *smox* were detected in multiple endocrine lineages, including β cells, δ cells, and α cells, as well as stromal populations (Fig. 1*D*). Together, these findings show that Smox is conserved, developmentally regulated, and its gene is expressed across pancreatic endocrine lineages, supporting its potential involvement in β-cell responses to inflammatory stress.

### Smox amplifies β-cell ROS generation and macrophage recruitment in a zebrafish model of β-cell injury

**Figure 2:**
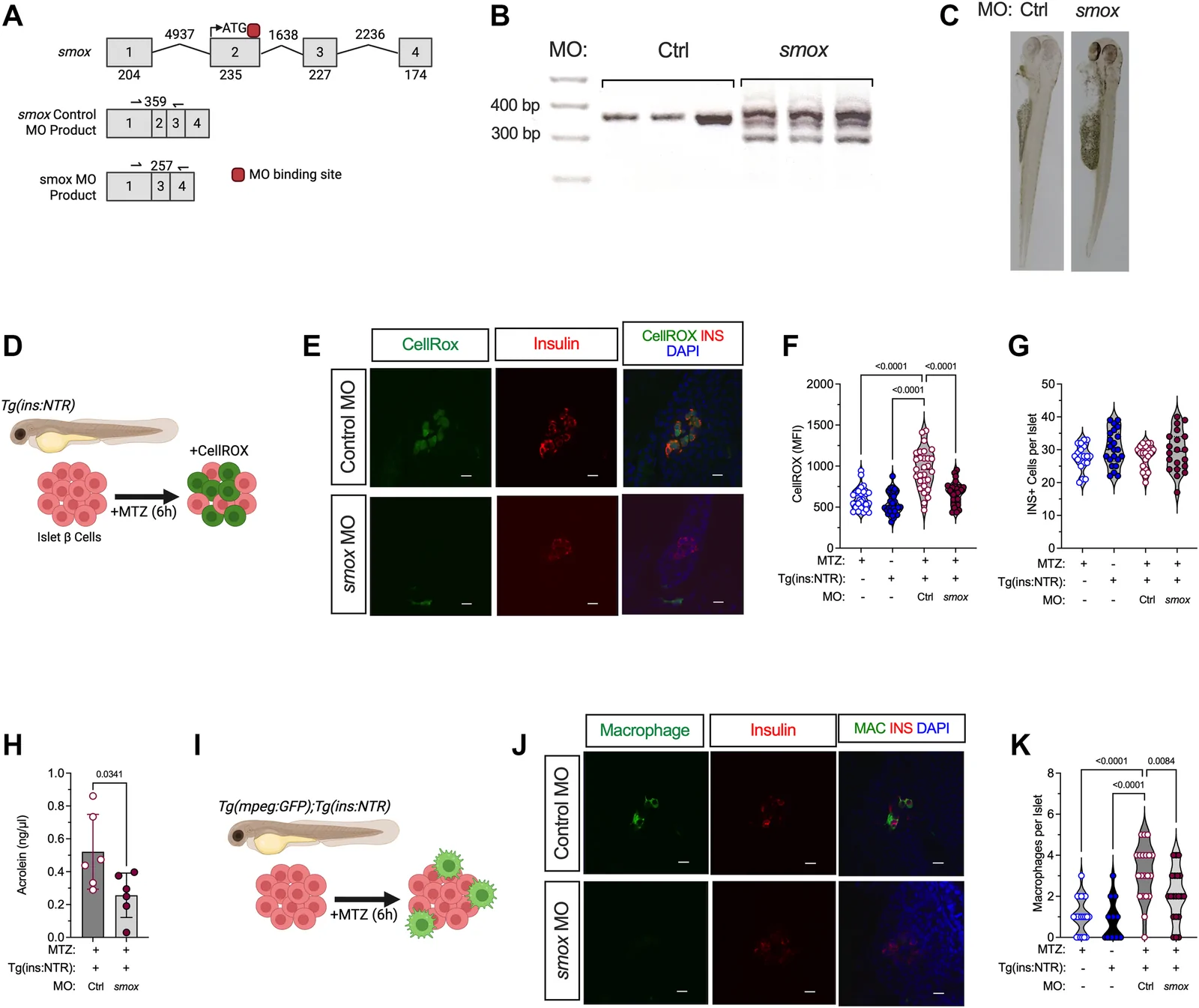
**Smox contributes to β-cell ROS generation and macrophage recruitment during MTZ-induced β-cell injury.***A*, diagram of *smox* exon–intron structure and splice-blocking morpholino target site, with predicted PCR products for control and *smox* morpholino injection. *B*, RT-PCR confirmation of *smox* gene splicing. *C*, Representative whole-body images of control and *smox* morpholino-injected zebrafish. *D*, schematic of the β-cell injury model using Tg(ins:NTR) embryos treated with MTZ for 6 h and assessed with the ROS-sensitive dye CellROX Green. *E*, representative images of CellROX (*green*), insulin (*red*), and DAPI (*blue*) staining in control MO– and *smoxMO*–injected embryos following 6 h MTZ exposure. Scale bars = 10 μm. *F*, quantification of CellROX mean fluorescent intensity (MFI) in insulin-positive β cells. N = 19 to 22 biological replicates. One-way ANOVA with Dunnett’s post-test. *G*, quantification of insulin-positive cells per islet. One-way ANOVA with Dunnett’s post-test. *H*, whole zebrafish acrolein levels. N = 5 to 6 pooled biological replicates. Two-tailed *t* test. *I*, schematic of macrophage recruitment assay in Tg(mpeg1:GFP);Tg(ins:NTR) embryos following 6 h MTZ exposure. *J*, representative images of macrophages (*green*), insulin (*red*), and DAPI (*blue*) in control- and *smox*MO–injected embryos. Scale bars = 10 μm. *K*, quantification of macrophage number per islet. N = 19 to 22 biological replicates. One-way ANOVA with Dunnett’s post-test. Data are presented as mean ± SEM.

To test Smox function *in vivo*, we designed a splice-blocking morpholino (*smoxMO*) that targets the splice acceptor site at the exon two-intron 2 junction (Fig. 2*A*). RT-PCR confirmed a reduction of correctly spliced transcript and showed aberrant products consistent with splicing disruption (Fig. 2*B*), and imaging confirmed that *smoxMO*-injected embryos exhibited preserved gross morphology relative to control MO-injected embryos (Fig. 2*C*). Although these imaging findings suggest that major developmental abnormalities are unlikely, they do not exclude more subtle developmental contributions to the observed injury phenotypes.

To determine whether Smox influences β-cell stress responses during inflammatory injury, we used the nitroreductase-metronidazole (NTR-MTZ) zebrafish model *Tg(ins:NTR*)^s950^, which enables temporally controlled β-cell–specific ROS generation. Our prior work demonstrated that MTZ treatment in this line produces an autonomous burst of oxidative stress that precedes overt β-cell death (13). Zygotes were injected with either a control morpholino or *smoxMO*, and then at 72 hpf, these embryos were exposed to 7.5 mM MTZ for 6 h (Fig. 2*D*). Consistent with our prior study, the use of this early injury time point preceded extensive cell loss, and β-cell number was comparable across treatment groups (Fig. 2, *E* and *F*). However, ROS levels, measured using the oxidation-dependent fluorophore CellROX, were markedly increased in β cells of MTZ-treated embryos that were injected with a control morpholino (Fig. 2, *E* and *G*). In contrast, embryos injected with *smoxMO* exhibited significantly reduced ROS accumulation, approaching levels seen in untreated or *Tg(ins:NTR)*-negative controls. Moreover, MTZ-injected embryos showed the expected reduction in acrolein, a product produced alongside hydrogen peroxide in the Smox reaction (Fig. 2*H*). These findings indicate that Smox activity contributes to ROS generation during β-cell injury.

Because β-cell oxidative stress promotes innate immune activation, we next assessed macrophage recruitment using *Tg(mpeg1:GFP);Tg(ins:NTR)* embryos, in which macrophages are marked by GFP expression (Fig. 2*I*). After the same 6 h MTZ exposure, control morpholino-injected embryos showed robust macrophage accumulation around the β cells, whereas embryos injected with the *smoxMO* displayed markedly diminished macrophage recruitment (Fig. 2, *J* and *K*). Together, these data support a model in which Smox promotes both ROS generation and early inflammatory cell engagement during β-cell injury.

### Knockdown of *smox* activates an alternative acetylation arm of the polyamine pathway

**Figure 3:**
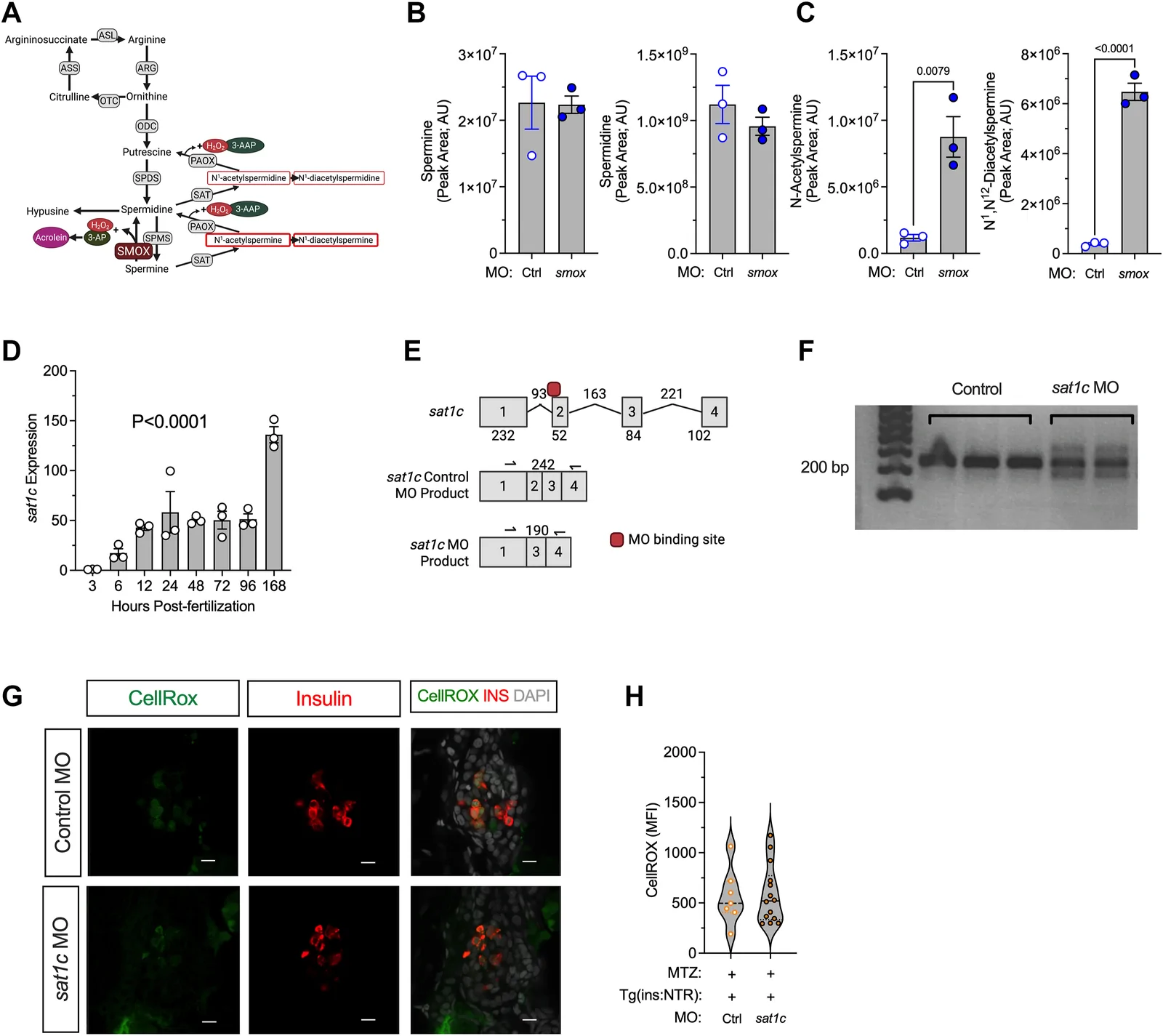
**SMOX knockdown redirects polyamine flux towards acetylated polyamine products.***A*, schematic of the polyamine pathway. *B*, spermine and spermidine levels in control- and *smox*MO–injected embryos. N = 3 biological replicates. Two-sided *t* test. *C*, n-acetylspermidine and N^1^,N^12^-diacetylspermine levels in control- and *smox*MO–injected embryos. N = 3 biological replicates. Two-sided *t* test. *D*, developmental expression of *sat1c* in whole zebrafish embryos as measured by quantitative PCR and normalized to *g6bp*. N = 3 biological replicates. *E*, diagram of *sat1c* exon–intron structure and splice-blocking morpholino target site, with predicted PCR products for control and *sat1c* morpholino injection. *F*, RT-PCR confirmation of *sat1c* gene splicing. *G*, representative images of CellROX (*green*), insulin (*red*), and DAPI (*blue*) staining in control- and *sat1c*MO–injected embryos following 6 h MTZ exposure. Scale bars = 10 μm. *H*, quantification of CellROX mean fluorescent intensity (MFI) in insulin-positive β cells. N = 7 to 14 biological replicates. Two-sided *t* test. Data are presented as mean ± SEM.

To further characterize the role of Smox in β-cell injury, we next assessed how the loss of Smox alters polyamine metabolism. Using mass spectrometry, we measured a panel of polyamines and metabolites involved in polyamine metabolic and catabolic pathways (Fig. 3*A*) in zebrafish embryos injected with either control or *smoxMO*. Zebrafish had quantifiable levels of each measured metabolite and polyamine, including bacterial-derived polyamines (Table 1). Whereas *smoxMO* injection did not alter levels of spermine and spermidine (Fig. 3*B*), it instead led to an increase in their respective acetylated species, N-acetylspermidine and N^1^,N^12^-diacetylspermine (Fig. 3*C*). This combination of stable spermine/spermidine levels together with elevated acetylated species is consistent with a compensatory shifting of the pathway: increasing flux through the Sat1/Paox arm.

**Table 1:** Polyamine and metabolite levels

Metabolite	Formula	Control ± SEM (Peak Area × 10^6^)	Smox MO ± SEM (Peak Area × 10^6^)	*p*-value
Acetylspermidine	C9H21N3O	190 ± 26	473 ± 47	**0.0008**
Arginine	C6H14N4O2	1053 ± 133	1217 ± 30	0.2947
Argininosuccinate	C10H18N4O6	7.5 ± 1.2	8.3 ± 1.6	0.5685
Citrulline	C6H13N3O3	42 ± 13	43 ± 1.6	0.9572
Dimethylarginine	C8H18N4O2	137 ± 40	181 ± 24	0.1835
Lysine	C6H14N2O2	652 ± 133	719 ± 33	0.4446
Ornithine	C5H12N2O2	150 ± 27	110 ± 13	0.0842
Putrescine	C4H12N2	31 ± 10	29 ± 3.8	0.7346
Spermidine	C7H19N3	1120 ± 249	957 ± 118	0.3601
Spermine	C10H26N4	23 ± 6.9	22 ± 2.2	0.9459
Acetylspermine	C12H28N4O	1.2 ± 0.4	8.8 ± 2.6	**0.0079**
1,3-Diaminopropane	C3H10N2	9.3 ± 1.8	7.6 ± 0.9	0.2263
Aminopropylcadaverine	C8H21N3	9.7 ± 4.1	11 ± 2.1	0.6854
Diacetylspermine	C14H30N4O2	0.4 ± 0.09	6.5 ± 0.6	**<0.0001**
Cadaverine	C5H14N2	7.9 ± 1.9	6.9 ± 1.3	0.4678
Hypusine	C10H23N3O3	1.3 ± 0.3	1.7 ± 0.09	0.0838

Bold values signify p values < 0.05.

### Sat1 does not contribute to β-cell ROS generation in a β-cell injury model

Because this parallel compensatory response would depend on Sat1 activity, we next examined the expression of zebrafish *sat1* paralogs. While *sat1a* and *sat1b* transcripts were not detected, *sat1c* expression was evident at three hpf, and its expression increased throughout the developmental stages examined through 168 hpf (Fig. 3*D*). To investigate *sat1c* functions, we designed a splice-blocking morpholino targeting the intron one-exon two splice junction (*sat1cMO*; Fig. 3*E*). RT-PCR of *sat1cMO*-injected animals confirmed the efficient reduction of correctly spliced transcript, and the appearance of additional products that indicate altered pre-mRNA splicing (Fig. 3*F*). We then asked if loss of Sat1, like the loss of Smox, altered β-cell ROS generation in the NTR–MTZ injury model. *Tg(ins:NTR)*^*s950*^ embryos injected with control or *sat1cMO* were treated with MTZ and stained with CellROX. Unlike Smox depletion, Sat1 depletion did not change β-cell ROS intensity (Fig. 3, *G* and *H*). These data indicate that although *sat1c* is expressed during pancreatic development, its acute reduction does not affect ROS generation in this model. Taken together, these findings show that loss of Smox shifts polyamine metabolism toward acetylated products without altering core polyamines, whereas loss of Sat1 alone does not modify ROS levels. This identifies Smox as the key enzyme linking polyamine catabolism to oxidative stress in this system.

### Smox drives cell cycle progression and stress-associated signaling pathways

**Figure 4:**
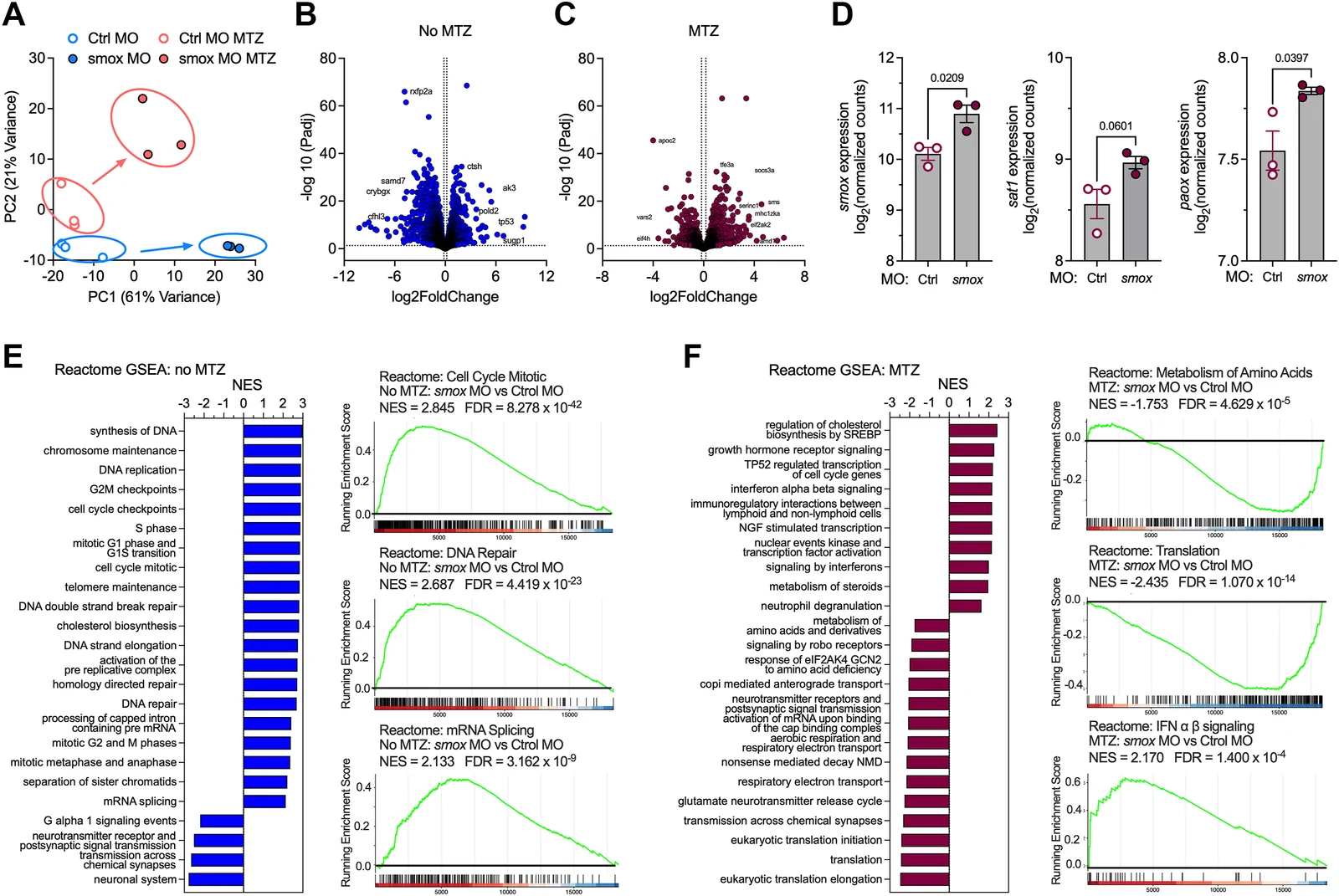
**SMOX knockdown alters transcriptional programs under basal conditions and during MTZ-induced β-cell injury.***A*, principal component analysis of RNA-seq data from control- and *smox*MO–injected embryos with or without MTZ treatment. *B*, Volcano plot showing differential gene expression between control- and *smox*MO–injected embryos in the absence of MTZ. *C*, Volcano plot showing differential gene expression between control- and *smox*MO–injected embryos following MTZ treatment. *D*, expression levels of *smox*, *sat1*, and *paox* transcripts measured by RNA-seq, shown as counts per million. Two-sided *t* test. *E*, reactome gene set enrichment analysis of control-vs *smox*MO–injected embryos in the absence of MTZ, with selected enrichment plots shown. *F*, reactome gene set enrichment analysis of control-vs *smox*MO–injected embryos following MTZ treatment, with selected enrichment plots shown. N = 3 pooled biological replicates.

To define how Smox activity shapes transcriptional responses under basal and injury conditions, we performed bulk RNA sequencing on whole *Tg(ins:NTR)*^*s950*^ zebrafish embryos at 72 hpf that had been injected with either control MO or *smoxMO* and had either been subjected to a 6 h MTZ injury or left untreated (all genes identified, their fold-changes, and statistical significance are provided in Table S1). Principal component analysis revealed clear segregation of samples by MTZ treatment and morpholino treatment (Fig. 4*A*). In the absence of MTZ, control MO- and *smoxMO-*injected samples formed distinct, non-overlapping clusters, indicating that loss of Smox induces a reproducible transcriptional shift under basal conditions. In addition, MTZ exposure alone produced a distinct separation between the control MO-injected samples along principal component axis 2. Most importantly, among the MTZ-treated samples, those injected with *smoxMO* segregated from those injected with control MO, demonstrating that Smox depletion significantly alters the transcriptional response to ROS-induced injury. Differential expression analysis further supported these findings. In the “non-MTZ” condition, comparison of *smoxMO versus* control MO revealed widespread transcriptional changes, with numerous genes exhibiting large and statistically significant fold changes (Fig. 4*B*). In the MTZ-treated condition, loss of Smox similarly resulted in robust differential gene expression relative to control MO-injected, indicating that Smox depletion alters gene expression both at baseline and in the context of β-cell injury (Fig. 4*C*). Consistent with the morpholino-mediated knockdown strategy, *smox* transcript levels were significantly increased in *smoxMO-*injected embryos, potentially reflecting compensatory mechanisms following functional inhibition (Fig. 4*D*). Expression of other polyamine catabolic enzymes were also altered, with *sat1b* and *paox* transcripts significantly elevated in *smoxMO* embryos, indicating coordinated remodeling of the polyamine metabolic network.

Pathway-level analysis revealed that the transcriptional programs affected by Smox depletion differed substantially between basal and injury conditions. In the absence of MTZ, Reactome GSEA showed strong enrichment of pathways related to cell cycle progression, DNA replication, chromosome maintenance, DNA repair, and mRNA splicing in *smoxMO*-injected samples, when compared with control MO-injected (Fig. 4*E*). These pathways exhibited high normalized enrichment scores and highly significant false discovery rates, demonstrating that loss of Smox drives coordinated upregulation of core proliferative and genome maintenance programs under basal conditions during development. In contrast, under conditions of MTZ-induced injury, Smox depletion significantly altered stress-responsive transcriptional networks (Fig. 4*F*). Reactome GSEA identified differential enrichment of pathways related to IFN-α and -β signaling, TP52-regulated transcription, amino acid metabolism, and translational initiation and elongation. Notably, pathways involved in protein synthesis and translation were negatively enriched, while immune and stress-associated signaling pathways were positively enriched, indicating a broad reprogramming of the injury response with loss of Smox activity.

Together, these data demonstrate that Smox depletion induces significant and biologically distinct transcriptional changes under both basal and injury conditions, establishing Smox as a regulator of both steady-state and injury-induced transcriptional programs.

## Discussion

Polyamines support essential β-cell functions, including nucleic acid stabilization, chromatin organization, modulation of ion channels, control of translation through hypusination of eIF5A, and adaptation to metabolic and oxidative stress (6, 17, 18, 19). At the same time, their catabolism *via* Smox and related enzymes generates hydrogen peroxide and reactive aldehydes, such as 3-aminopropanal and acetylated aminopropanal, which can exacerbate β-cell dysfunction (7, 8, 9). This dual nature of polyamine biology raises important questions about which components of the pathway are beneficial and which amplify injury, under varying conditions. In this context, our study identifies Smox as a key metabolic node in zebrafish whose activity shapes the β-cell response to oxidative and inflammatory stress. In the gastrointestinal tract, studies have shown that induction of the Smox protein during *H. pylori* infection promotes oxidative DNA damage and inflammation, contributing to epithelial injury and carcinogenesis (20, 21). Initial studies in *Smox* knockout mice provided evidence in mammalian systems that the loss of Smox alters susceptibility to inflammatory and toxic injury (22, 23). These findings establish Smox as a conserved mediator of stress-associated tissue injury and provide an important context for interpreting its role in metabolically stressed β cells.

The high degree of structural and sequence conservation between zebrafish and mammalian Smox orthologs supports a conserved biological role for this enzyme. Furthermore, publicly available *in situ* hybridization data from the ZFIN database further support pancreatic expression of *smox* during zebrafish development beginning at 19 hpf, consistent with the transcriptional analyses presented here and suggesting that *smox* is positioned to influence endocrine pancreas biology. Prior studies have shown that enzymes in the polyamine and stress response network are induced in β cells or islets exposed to inflammatory or metabolic stressors, supporting the idea that these pathways are part of a broader adaptive stress-responsive machinery (13). Our developmental and comparative analyses are consistent with this concept and justify the use of zebrafish models to probe how Smox activity influences β-cell injury responses *in vivo*. To identify roles for Smox, we selected a time point in our β-cell injury model when there was no difference in β-cell number between control and MTZ-treated animals; this approach allowed us to examine β-cell ROS levels independently of cellular loss. Under these conditions, Smox-deficient embryos were markedly resistant to the induction of oxidative stress. In the Tg(ins:NTR) model, MTZ metabolism initiates an early burst of oxidative injury in β cells, independent of polyamine metabolism. We propose that this initial insult subsequently engages secondary stress pathways, including Smox-dependent polyamine catabolism, thereby further amplifying oxidative stress by generating hydrogen peroxide and reactive aldehydes. Thus, Smox depletion does not prevent the initiation of the NTR-MTZ reaction but instead attenuates the downstream accumulation of oxidative stress and associated inflammatory responses.

The reduction in ROS with *smoxMO* in our zebrafish model is consistent with the known biochemical consequences of Smox activity, whereby it converts spermine to spermidine while generating hydrogen peroxide and reactive aldehydes, including 3-aminopropanal and acrolein (8, 9). Spermine can also be metabolized through the Sat1-Paox pathway, which produces N-3-acetylaminopropanal (3-AAP) and hydrogen peroxide, but does not generate acrolein. Our studies, however, showed that the loss of Sat1 did not affect ROS generation in response to MTZ-induced injury. At face value, these findings suggest that the contribution of Smox to ROS generation may primarily lie in acrolein generation rather than hydrogen peroxide production alone. Acrolein has been shown to form deleterious stable adducts with cellular proteins, impair mitochondrial function, and disrupt membrane integrity, all of which could heighten cellular ROS accumulation and vulnerability (9).

Our polyamine metabolomics data provide additional mechanistic insight into how Smox deficiency in zebrafish might protect against ROS production in β cells. Notably, we found that Smox-deficient zebrafish accumulate acetylated polyamines, including N-acetylspermidine and N^1^,N^12^-diacetylspermidine, while maintaining pools of spermidine and spermine at similar levels. This pattern is consistent with established models of polyamine metabolic regulation (24, 25) and may indicate a rerouting of polyamine flux *via* Sat1-dependent acetylation pathways, suggesting a compensatory metabolic response to prevent depletion of primary polyamine pools. Increased Sat1 activity is known to promote polyamine turnover through acetylation and export, allowing continued pathway flux when primary catabolic routes are altered (6, 7, 8). Therefore, the elevation of acetylated polyamine levels observed here is most consistent with compensatory flux *via* the Sat1 arm and their subsequent accumulation due to inadequate Paox activity. However, whether this metabolic adaptation contributes directly to the protective phenotype remains unknown. Although acute Sat1c depletion alone did not alter ROS accumulation, these studies do not determine whether Sat1c activity becomes necessary under conditions of Smox deficiency. Future studies employing combined perturbation of these pathways will be required to establish whether compensatory acetylation contributes functionally to protection. Our data suggest that, in the absence of Smox, shunting of spermine *via* the alternative Sat1-Paox pathway is incomplete, resulting in an overall reduction in the generation of hydrogen peroxide and acrolein. This predominance of the Smox pathway to generate hydrogen peroxide and reactive aldehydes has also been observed in some mammalian cells (8, 26).

Increasing spermine synthase activity might be viewed as a counterbalance to enhanced Smox activity. Higher Sms activity could expand intracellular spermine pools, potentially enhancing the antioxidant and cytoprotective functions of spermine. Conversely, increased spermine availability could also increase substrate flux through Smox, thereby augmenting the generation of hydrogen peroxide and reactive aldehydes under conditions of elevated Smox activity. Thus, the effects of Sms hyperactivity would likely depend on the balance between polyamine synthesis and catabolism and cannot be predicted from the present data.

In parallel with reduced oxidative stress, Smox depletion led to a pronounced decrease in macrophage accumulation around the injured principal islet in our studies. Although our study does not focus on inflammatory disease mechanisms *per se*, this observation is consistent with the established link between oxidative stress signals and recruitment of innate immune cells. Aldehyde adducts, oxidized lipids, ROS-modified proteins, and related metabolites can form a microenvironment that attracts phagocytic cells and shapes macrophage behavior (13, 27, 28). Thus, the decrease in macrophage infiltration observed with the loss of Smox is likely a downstream consequence of reduced injury-associated signaling rather than a difference in the degree of β-cell ablation. However, because *smoxMO* was delivered systemically, the reduced macrophage response cannot be assigned solely to Smox activity in β cells. Smox depletion in macrophages or other cell types may independently alter their recruitment or migration. Tissue-specific genetic models will be required to determine whether the observed phenotype arises from β cells, immune cells, or both.

Transcriptional analyses further extend our mechanistic understanding by demonstrating that Smox activity shapes both baseline cellular programs and the nature of the response to injury. In the absence of MTZ, Smox deficiency induces a transcriptional shift characterized by increased expression of genes in pathways related to cell cycle progression, DNA replication and repair, and RNA processing. These changes are consistent with established roles of polyamine metabolism and hypusination in regulating proliferation, genome maintenance, and translational capacity (6, 7, 17, 29, 30). Importantly, this baseline transcriptional state shows no enrichment of injury-responsive pathways, indicating that loss of Smox primarily alters homeostatic programs rather than placing cells in a stressed or damaged state. By contrast, under MTZ-induced β-cell injury, Smox depletion reprograms the injury response and affects pathways governing translation, amino acid metabolism, mitochondrial respiration, and interferon-associated signaling. Suppression of translational initiation and elongation, together with altered amino acid metabolic programs, is consistent with activation of stress-responsive pathways that are sensitive to protein damage and metabolic imbalance, including those regulated through integrated stress response signaling (18, 31, 32). Concurrent enrichment of interferon and immune-associated transcriptional programs aligns with prior work showing that cellular stress within β cells can propagate inflammatory signaling without directly reporting the extent of cell loss (33, 34). When considered alongside the metabolomic and phenotypic data, these transcriptional changes support a model in which Smox-derived metabolites amplify secondary stress signaling during injury.

Some limitations of our study should be acknowledged. Our studies were restricted to zebrafish and cannot be directly extrapolated to mammalian diabetes models. Nevertheless, the conservation of Smox across disparate species both justifies and invites impactful future studies interrogating tissue-specific gene inactivation in mice. Because *smox* depletion was achieved systemically at the zygote stage, developmental contributions and potential off-target effects cannot be entirely excluded. Although *smoxMO*-injected embryos exhibited preserved gross morphology and pancreatic architecture, future studies employing inducible and cell type-specific genetic approaches will be required to establish gene specificity and distinguish developmental from injury-specific functions. In addition, polyamine metabolite measurements were performed on whole-zebrafish lysates, thereby limiting our ability to determine tissue- or β-cell-specific changes in polyamine flux. Also, our study did not directly interrogate the consequences of ROS accumulation or attenuation on β-cell function, a challenge in zebrafish embryos. The implications of β-cell ROS attenuation have been studied in mammalian systems previously and have been shown to improve β-cell insulin secretion and reduce diabetes severity (35, 36, 37). Taken together, our findings suggest that Smox activity influences how β cells interpret and respond to oxidative injury signals. (7, 8, 9). Depleting Smox preserves polyamine-dependent homeostatic functions while limiting activation of maladaptive stress and immune signaling pathways, providing a transcriptional correlate of reduced oxidative stress and immune cell recruitment.

## Experimental procedures

### Sequence alignment

The phylogenetic tree was constructed using the Ensembl Gene Orthology/Paralogy prediction method. Trees were assembled using the orthologous protein for every species and longest translation by CCDS was used. Taxonomy IDs refer to the NCBI Taxonomy Browse, and multiple alignments were made using MUSCLE (38).

### Zebrafish maintenance and treatment

All zebrafish studies were performed under protocols approved by the University of Chicago Institutional Animal Care and Use Committee. Wild-type (AB), *Tg(ins:Flag-NTR)*^*s950*^ (ZDB-ALT-130930-5) (39), and *Tg(mpeg1:GFP)* (ZDB-ALT-120117-1) zebrafish were maintained at 28.5 °C in a recirculating aquaculture system and maintained under a 14-/10-h light/dark cycle. Heterozygous outcrossed embryos bearing *Tg(ins:Flag-NTR)*^*s950*^ were collected at spawning and maintained in a 28.5 °C incubator in egg water-filled petri dishes. Transgenic zebrafish embryos were genotyped by epifluorescence at 80 hpf using an Olympus MVX10 stereo dissecting microscope. 1-Phenyl-2-thiourea (PTU; Acros #207250250) supplementation at 0.003% was used to prevent pigmentation in all embryos after gastrulation stages. For induction of oxidative stress, a 7.5 mM metronidazole (MTZ; Sigma #095K093) solution was prepared in egg water (0.1% instant ocean salt, 0.0075% calcium sulfate) that was supplemented with PTU.

### Zebrafish RT-PCR, morpholino design, and genotyping

For quantitative expression analyses, total mRNA was extracted from 25 embryos per sample at each stage using Trizol/Chloroform extraction as previously described (40). Isolated RNA was measured using a nanodrop (Thermo Scientific) and reverse-transcribed to cDNA using a High-Capacity cDNA Reverse Transcription kit (Applied Biosystems 4388950). SYBR green based quantitative RT-PCR was analyzed across time points as previously described (14), with *smox* products normalized to *actb* and *sat1c* products normalized to *g6pd* using the following primers: *smox.2* F: GCCTTGCTGCTACCAAAACC; *smox.2* R: TGGAAGAACTCCTGAGTCAGC; *actb.1* F: CGAGCAGGAGATGGGAACC*; actb.1* R: CAACGGAAACGCTCATTGC; *sat1c.2* F: AAATGGCCAAGTTCATATTACGG*; sat1c.2* R: AGTGAAGTAGTACATGGCAAAGC; *g6pd.1* F: GTCCCGAAAGGCTCCACTC; and *g6pd.1* R: CCTCCGCTTTCCTCTC.

Standard control (5′-CCTCT TACCT CAGTT ACAAT TTATA-3′), *smox* morpholino (*smox* MO; 5′-AAAGG AAGCT GTTGT CTCAC CGTGC-3′), and sat1c morpholino (*sat1c* MO; 5′-GTTCCTGATATGATTTCCATCACGT-3′) oligonucleotides were obtained from GeneTools, LLC, dissolved in molecular grade water, and injected into zebrafish zygotes prior to the 8-cell embryo stage using a uPUMP micro-injector (World Precision Instruments) as previously described (41).

For morpholino confirmation experiments, total mRNA was extracted at 48 hpf, and *smoxMO* samples were run with the primers described above. mRNA from *sat1MO* was run with the following primers: F: AAATGGCCAAGTTCATATTACGG and R: AGTGAAGTAGTACATGGCAAAGC. PCR products were run on a 2.5% agarose gel at 110V for an hour before visualization.

### Zebrafish RNA isolation and sequencing

Total RNA was extracted from 20 zebrafish embryos/sample at 6 h post-MTZ treatment through Trizol/chloroform extraction as described above (40). Reads were aligned to the GRCz11 genome build, and sequencing was performed by Innomics using the DNBSEQ platform and subsequent analysis. Transcripts were summarized to the gene level using tximport (v1.30.0), and the resulting counts matrix was analyzed using DESeq2 (v1.42.1) in R. Counts normalization was conducted *via* variance stabilization transformation (vst), and the normalized counts were used for principal component analysis and heatmap visualizations. The transcripts were summarized to the gene level using tximport (v.1.30.0), and the resulting counts matrix was analyzed using DESeq2 (v1.42.1) in R. Genes with raw counts greater than 10 in at least three samples were kept for downstream analyses. Counts normalization was conducted *via* variance stabilization transformation (vst), and the normalized counts were used for principal component analysis and heatmap visualizations. The counts were modeled by the design formula ∼ Full_Treatment, which combined the factors of interest (vehicle, MTZ, control morpholino, and Smox morpholino) into a single factor for differential expression testing. The Benjamini-Hochberg method was used for *p*-value adjustment, and genes with adjusted-*p*-values < 0.05 and an absolute fold change >1.5 were considered differentially expressed. Pathway analysis was performed using Gene Ontology (GO) and gene set enrichment analysis (GSEA). Genes were pre-ranked using the Wald statistic and used as input for GSEA implemented by clusterProfiler (v4.10.1). Hallmark Pathway and Reactome gene sets were retrieved from the Molecular Signatures Database (msigdbr, v25.1.1).

### Acrolein ELISA

Acrolein was detected using the acrolein (ACR) ELISA Kit (MyBioSource; #MBS7213206). 25 embryos per sample were collected at 72 hpf in PBS using a tissue homogenizer. Samples were diluted using 1:1 of the PBS homogenate and the assay procedure according to kit instructions. Concentration of samples were calculated using a 4PL non-linear regression model for best fit.

### Polyamine metabolomics

For metabolite profiling, 25 zebrafish embryos were euthanized and collected at 72 hpf and immediately flash frozen. Frozen embryos were extracted with ice-cold 4/4/2 acetonitrile/methanol/water containing 0.1% formic acid (20 μl solvent/mg of embryos), vortexed, followed by two times sonication and freeze–thaw cycles. Then samples were incubated on ice for 20 min, centrifuged for 30 min at 4 °C and 18,000*g* and 100 μl of supernatant was dried down using Genvevac EZ-2 4.0 elite evaporator and stored at-80 °C. Samples were re-suspended in 50 μl of 60/40 acetonitrile/water on the day of analysis. All solvents were LC-MS grade and obtained from Fisher Scientific. Polyamines separation was performed using Thermo Scientific Vanquish Horizon UHPLC system and Atlantis BEH Z-HILIC (2.1 × 150 mm, 2.5 μM; Waters Corporation) column at acidic pH. The mobile phase A (MPA) was 10 mM ammonium formate containing 0.2% formic acid and mobile phase B (MPB) was acetonitrile containing 0.1% formic acid. The column temperature, injection volume, and flow rate were 30 °C, 5 μl, and 0.2 ml/min, respectively. The chromatographic gradient was 0 min: 90% B, 15 min: 20% B, 16 min: 20% B, 16.5 min: 90% B, 17 min: 90% B, and 23 min: 90% B. During the re-equilibration, the flow rate was increased to 0.4 ml/min for 4.7 min. MS detection was done using Orbitrap IQ-X Tribrid mass spectrometer (Thermo Scientific) with a H-ESI probe operating in switch polarity mode for both methods. MS parameters were as follows: spray voltage: 3800 V for positive ionization and 2500 V for negative ionization modes, sheath gas: 80, auxiliary gas: 25, sweep gas: 1, ion transfer tube temperature: 300 °C, vaporizer temperature: 300 °C, automatic gain control (AGC) target: 25%, and a maximum injection time of 80 milliseconds (ms). The data acquisition was done using the Xcalibur software (Thermo Scientific) in full-scan mode with a range of 70 to 600 m/z at 120K resolution. Polyamines were identified by matching the retention time and MS/MS fragmentation to the reference standards. Data analysis was performed using Skyline (Adams *et al.*, 2020).

### Reactive oxygen species (ROS) staining

Cellular ROS was detected using CellROX green reagent (Invitrogen #C10444) and lipid peroxides by 4HNE (Invitrogen MA5-27570); live embryos were transferred to 1.5 ml microcentrifuge tubes, washed with egg water, then incubated in the dark for 1 h at 28.5°C with 10 μM CellROX green diluted in egg water. At the conclusion of each experiment, embryos were washed in egg water then fixed with 3% formaldehyde in PEM buffer (0.21 M PIPES, 1 mM MgSO4, 2 mM EGTA, and pH 7) at 4 °C overnight. Fixed embryos were washed with PBS and deyolked, then antibody staining was performed as described (13) with guinea pig anti-insulin (1:200; Invitrogen #180067) and detected with complementary Alexa-conjugated secondary antibodies (1:500; Jackson ImmunoResearch). DNA was stained with TO-PRO3 (ThermoFisher #T3605) diluted 1:2000. After staining, embryos were mounted on slides in VECTASHIELD (Vector Labs H-1000), and confocal imaging was performed with a Nikon microscope.

### Statistical analyses

For imaging experiments, each data point represents one embryo unless otherwise stated. Embryos were pooled from independent clutches for three biological replicates per condition. For metabolomics and RNA sequencing, each biological replicate consisted of an independently collected pool of 20 and 25 embryos respectively. Exact sample sizes are provided in the figure legends. For comparisons involving two conditions, a 2-tailed student’s unpaired *t* test was performed. For comparisons involving >2 conditions, a one-way ANOVA was performed. GraphPad Prism, version 10, was used for these statistical analyses and corresponding visualizations. Statistical significance was defined as *p* < 0.05.

## Data availability

Some of the data generated and analyzed in the current study are not publicly available but can be obtained from the corresponding author upon reasonable request. RNA sequencing data have been uploaded to the public repository Gene Expression Omnibus (https://www.ncbi.nlm.nih.gov/geo/) with accession number GSE328689.

## Supporting information

This article contains supporting information.

## Conflict of interest

The authors declare that they have no conflicts of interest with the contents of this article.
