# Supplementary material for: Spermine oxidase promotes oxidative stress during islet inflammation
Source: J Biol Chem. 2026 Aug 7;302(9):113412. doi: 10.1016/j.jbc.2026.113412 (PMC13553577; doi:10.1016/j.jbc.2026.113412)
Supplement: Supplementary file 1 [file mmc1.docx]

**Supplemental Table 1. Complete differential gene expression analyses of zebrafish embryos following Smox depletion and MTZ-induced injury.** DESeq2 output is provided for the following pairwise comparisons: (1) vehicle-treated smoxMO versus vehicle-treated control MO embryos, (2) MTZ-treated control MO versus vehicle-treated control MO embryos, and (3) MTZ-treated smoxMO versus MTZ-treated control MO embryos. For each comparison, the table includes gene symbol, Entrez Gene ID, normalized mean expression (baseMean), log₂ fold change, log₂ fold change standard error (lfcSE), Wald test statistic, raw *P* value, Benjamini-Hochberg adjusted *P* value (padj), and unshrunk log₂ fold change estimates (lfcnoshrink). Differential expression analyses were performed using DESeq2 on RNA-sequencing data obtained from independently collected biological replicates of pooled zebrafish embryos.
